# Targeted sequencing of cancer‐related genes in nasopharyngeal carcinoma identifies mutations in the TGF‐β pathway

**DOI:** 10.1002/cam4.2429

**Published:** 2019-07-22

**Authors:** An‐Ko Chung, Chun‐Nan OuYang, Hsuan Liu, Mei Chao, Ji‐Dung Luo, Cheng‐Yang Lee, Yen‐Jung Lu, I‐Che Chung, Lih‐Chyang Chen, Shao‐Min Wu, Ngan‐Ming Tsang, Kai‐Ping Chang, Cheng‐Lung Hsu, Hsin‐Pai Li, Yu‐Sun Chang

**Affiliations:** ^1^ Graduate Institute of Biomedical Sciences Chang Gung University Taoyuan City Taiwan, Republic of China; ^2^ Molecular Medicine Research Center Chang Gung University Taoyuan City Taiwan, Republic of China; ^3^ Department of Biochemistry Chang Gung University Taoyuan City Taiwan, Republic of China; ^4^ Division of Colon and Rectal Surgery Chang Gung Memorial Hospital Taoyuan City Taiwan, Republic of China; ^5^ Department of Microbiology and Immunology Chang Gung University Taoyuan City Taiwan, Republic of China; ^6^ Liver Research Center, Department of Hepato‐Gastroenterology Chang Gung Memorial Hospital Taoyuan City Taiwan, Republic of China; ^7^ Bioinformatics Center Chang Gung University Taoyuan City Taiwan, Republic of China; ^8^ Research Information Session, Office of Information Technology Taipei Medical University Taipei City Taiwan, Republic of China; ^9^ ACT Genomics, Co. Ltd. Taipei City Taiwan, Republic of China; ^10^ Department of Medicine Mackay Medical College New Taipei City Taiwan, Republic of China; ^11^ Department of Radiation Chang Gung Memorial Hospital Chang Gung University Taoyuan City Taiwan, Republic of China; ^12^ Department of Otolaryngology‐Head and Neck Surgery Chang Gung Memorial Hospital Chang Gung University Taoyuan City Taiwan, Republic of China; ^13^ Division of Hematology‐Oncology, Department of Internal Medicine Chang Gung Memorial Hospital Chang Gung University Taoyuan City Taiwan, Republic of China

**Keywords:** copy number variation, mutation, nasopharyngeal carcinoma, next‐generation sequencing, TGF‐β signaling

## Abstract

Approximately, 25% of nasopharyngeal carcinoma (NPC) patients develop recurrent disease. NPC may involve relatively few genomic alterations compared to other cancers due to its association with Epstein‐Barr virus (EBV). We envisioned that in‐depth sequencing of tumor tissues might provide new insights into the genetic alterations of this cancer. Thirty‐three NPC paired tumor/adjacent normal or peripheral blood mononuclear cell samples were deep‐sequenced (>1000×) with respect to a panel of 409 cancer‐related genes. Newly identified mutations and its correlation with clinical outcomes were evaluated. Profiling of somatic mutations and copy number variations (CNV) in NPC tumors identified alterations in RTK/RAS/PI3K, NOTCH, DNA repair, chromatin remodeling, cell cycle, NF‐κB, and TGF‐β pathways. In addition, patients harbored CNV among 409 cancer‐related genes and missense mutations in TGF‐β/SMAD signaling were associated with poor overall survival and poor recurrence‐free survival, respectively. The CNV events were correlated with plasma EBV copies, while mutations in TGFBR2 and SMAD4 abrogate SMAD‐dependent TGF‐β signaling. Functional analysis revealed that the new TGFBR2 kinase domain mutants were incapable of transducing the signal, leading to failure of phosphorylation of SMAD2/3 and activation of downstream TGF‐β‐mediated cell growth arrest. This study provides evidence supporting CNV and dysregulated TGF‐β signaling contributes to exacerbating the NPC pathogenesis.

**Abbreviations**
CCND1: Cyclin D1
CCP: Comprehensive Cancer Panel
CDKN2A: cyclin‐dependent kinase inhibitor 2A
CDKN2B: cyclin‐dependent kinase inhibitor 2B
CNV: copy number variations
EBV: Epstein‐Barr virus
NPC: nasopharyngeal carcinoma
PBMC: peripheral blood mononuclear cells
PI3K: phosphatidylinositol‐3‐kinase
WES: whole‐exome sequencing
WT: wild‐type

## 1 INTRODUCTION

Nasopharyngeal carcinoma (NPC), which is a tumor arising from the nasopharynx epithelium, is highly prevalent in Southeast Asia, including southern China, Hong Kong, and Taiwan. NPC is closely associated with infection of the human herpesvirus, Epstein‐Barr virus (EBV).^1^ EBV‐infected cells express viral oncogenes that induce cell transformation. EBV latent and lytic genes can cause chromosomal instability.^2^, ^3^, ^4^, ^5^, ^6^ As seen in many cancers, multiple genetic alterations are present in NPC. Early studies identified the losses of chromosomes 3p and 9p as early events in the development of NPC.^7^, ^8^ Chromosome 3p21.3 was the first region to be functionally identified as a tumor suppressor gene (TSG) cluster,^9^ and 9p deletion results in the loss of the genes encoding the tumor‐suppressive cyclin‐dependent kinase inhibitor INK4 family members, p16 (*CDKN2A*) and p15 (*CDKN2B*). On the other hand, various groups studying NPC identified amplification of cyclin D1 (*CCND1*), *PIK3CA*, *AKT2*, *JAK2*, and *MYC*.^10^, ^11^, ^12^, ^13^ Several whole‐exome/genome sequencing studies on the mutational landscape of NPC revealed multiple genetic defects related to chromatin modification and the signaling pathways involving ErbB‐phosphatidylinositol‐3‐kinase (PI3K) and NF‐κB.^12^, ^14^, ^15^ In addition, mutation of tumor antigen p53 (*TP53*) is frequently seen in NPC, with reported prevalences ranging from 7.3% (9/124)^15^ to 8.5% (11/128).^12^ In most of the available reports, the sequencing depth of the whole‐exome sequencing (WES) was 30‐200×. Considering the heterogeneity of tumor cells in a given biopsy, we herein sought to identify yet‐unreported somatic mutations in Taiwanese NPC biopsies. Toward this end, we collected samples and performed targeted high‐depth sequencing using the Ion AmpliSeq^TM^ Comprehensive Cancer Panel (CCP), which targets 409 known oncogenes and TSGs.

From among the 409 cancer‐related genes, we identified 44 that had somatic mutations in our samples. Among them, mutations of the TGF‐β signaling‐associated genes, *TGFBR2* and *SMAD4*, were the most commonly mutated genes in our samples. Our present analyses revealed that the identified mutations in TGFBR2 and SMAD4 attenuated canonical TGF‐β‐mediated SMAD signaling, and the former conferred resistance to TGF‐β‐induced cell growth inhibition and cell cycle arrest. Furthermore, patients harboring the identified mutations in *TGFBR2* and *SMAD4* tended to have poor recurrence‐free survival. Together, our results indicate that dysregulation of SMAD‐dependent TGF‐β signaling may play an important role in the pathogenesis of NPC.

## 2 MATERIALS AND METHODS

Specimens and DNA extraction, next‐generation sequencing and analysis, validation of mutations by Sanger sequencing and pyrosequencing, validation of copy number variations (CNVs) by quantitative real‐time PCR, plasmids, immunoblot analysis, luciferase assay, immunofluorescence staining, lentiviral production and transduction, cell proliferation assay, flow cytometric analysis of cell cycle, animal tumor models, and statistical analysis can be accessed in the supporting information (Doc S1).

## 3 RESULTS

### 3.1 Deep‐sequencing results of 409 cancer‐related genes in NPC

To uncover novel somatic mutations in NPC, we performed deep‐targeted sequencing for all exons of 409 cancer‐related genes using predesigned primers from a CCP (Thermo Fisher Scientific, Table S1). Our samples consisted of 33 matched tumor and peripheral blood mononuclear cell (PBMC) or adjacent samples from NPC. Sequencing analysis of the targeted amplicons obtained from the 66 samples showed that the read depth had an average coverage of 1645× ± 396× (Table S2). The percentage of reads on targets and the uniformity of the read coverage for all samples are shown in Table S2. We identified 60 variants within 49 genes according to deep‐sequencing analysis. A detailed workflow of the DNA sequencing procedure and number of variants identified are summarized in Figure S1. The most common somatic mutation found in our NPC samples was the C > T transition (58%), which likely reflects the spontaneous deamination of 5‐methyl‐cytosine (Figure S2). The second most frequent signature in NPC was the C > A transversion (16% in Figure S2), which is associated with cigarette smoke exposure.^16^ This finding is consistent with previous results obtained from exome sequencing of NPC.^14^, ^15^, ^17^

To validate the 60 variants (49 genes) identified by deep sequencing, we chose NPC tumor samples with sufficient DNA amount and validated 43 variants by performing Sanger sequencing first and if the mutated sequence could not be detected by Sanger sequencing, we then choose pyrosequencing for alternative validation method using the indicated primers (Table S3). Among the 43 variants, 38 of them were confirmed either by Sanger sequence or pyrosequencing as listed in Table S4. Therefore, the validation rate was 38/43 (88%) (Table S4 and Figure S3). After we excluded the five variants that could not be validated, the remaining variants became 55 variants (44 genes). Interestingly, one NPC patients (NPC_003T) had two point mutations or variants in TP53.

### 3.2 Profiling of somatic mutations in NPC patients

**Figure 1:**
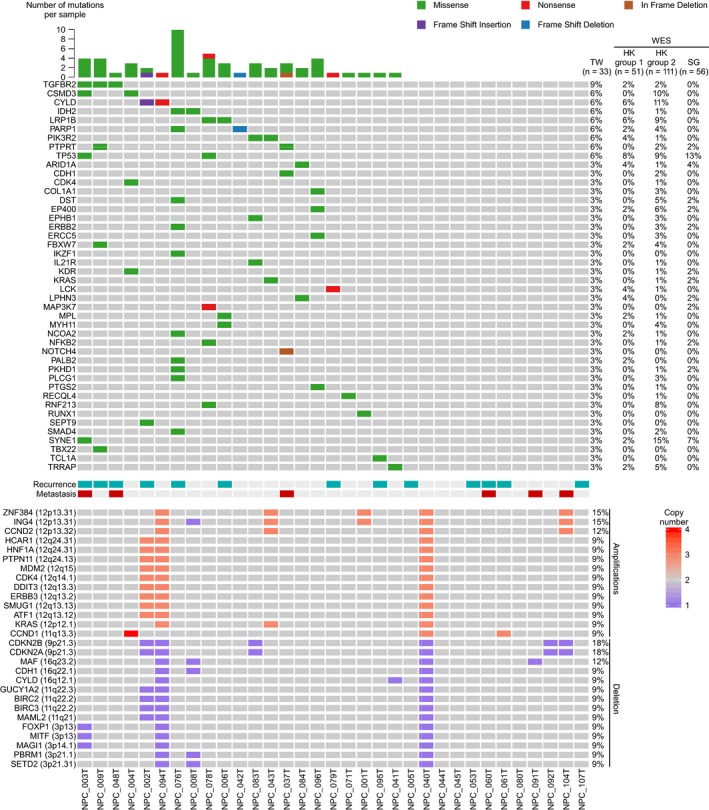
Genetic alterations profile of 33 NPC tumors. Matrix showing the gene alterations (rows) of 33 NPC tumors (columns) analyzed by targeted sequencing of 409 cancer‐associated genes. Gene mutations and CNVs were shown in upper panel and lower panel, respectively. Genes were ordered by mutation frequency and alphabet. The number of mutations for each patient was plotted in the upper panel. In comparison, the mutation frequency of the same gene in other WES studies (HK group 1, HKU; HK group 2, CUHK; and Singapore) was indicated. Each CNV shown in this figure was identified in at least three patients. Amplification and deletion are defined as copy number ≥3 and ≤1, respectively. The clinical outcomes (recurrence and metastasis) for each patient were indicated in the middle panel

Overall, we identified 55 nonsynonymous mutations in 21 of 33 (64%) samples; these mutations included 49 missenses, three stop‐gains, and three indels in 44 genes (Table S4.). Within 21 samples, each NPC sample had 1 to 10 mutations. Mutations in top ranked genes, *TGFBR2*, *CSMD3*, *CYLD*, *IDH2*, *LRP1B*, *PARP1*, *PIK3R2*, *PTPRT*, and *TP53* (Figure 1) were detected in at least two of the 33 samples and these relatively frequent mutations were identified in 14 of 33 (42%) tumors. It is of note that *TGFBR2* mutation was detected in three samples. Among them, *CSMD3* and *LRP1B* were reported as potential TSGs.^18^, ^19^ *PTPRT* encoding a tyrosine phosphatase to dephosphorylates STAT3 and paxillin may function as tumor suppressor.^20^ Mutations in *IDH2* and *PIK3R2* are reported to confer gain‐of‐function in cancer cells.^21^, ^22^ Using the six variant effect prediction tools, we observed that 11 mutations in *IDH2* (P151H), *TP53* (P180S and M207I), *SMAD4* (R361H), *PIK3R2* (D349N), *RUNX1* (P176S), *TGFBR2* (G253V and E290K), *PKHD1* (G584D), and *TBX22* (R151L) are predicted as deleterious mutations. Besides, 11 of the 55 nonsynonymous mutations were previously reported in the COSMIC database (Table S4). For example, KRAS (G12D) 23 is hotspot gain‐of‐function mutation. Three TP53 mutations (R174Q, P180S, and M207I) are all localized in the TP53 DNA‐binding domain (~102‐292 aa) and function to counteract p53‐mediated G1/S arrest.^24^ The SMAD4 (R361H) loss‐of‐function mutation results in a loss of heterocomplex formation and failure to mediate SMAD‐dependent TGF‐β responses.^25^ The mutation identified in the chromatin remodeler, ARID1A (G2087E) and ubiquitin ligase, FBXW7 (Y519C) is located in LXXLL motif (~2085‐2089 aa), which is reportedly involved in the interaction with nuclear hormone receptors,^26^ and WD40 domain, which is responsible for forming the β‐propeller surface that interacts with substrates for phosphorylation‐dependent ubiquitination,^27^ respectively. The above described mutations are considered as potential driver mutation in NPC.

**Figure 2:**
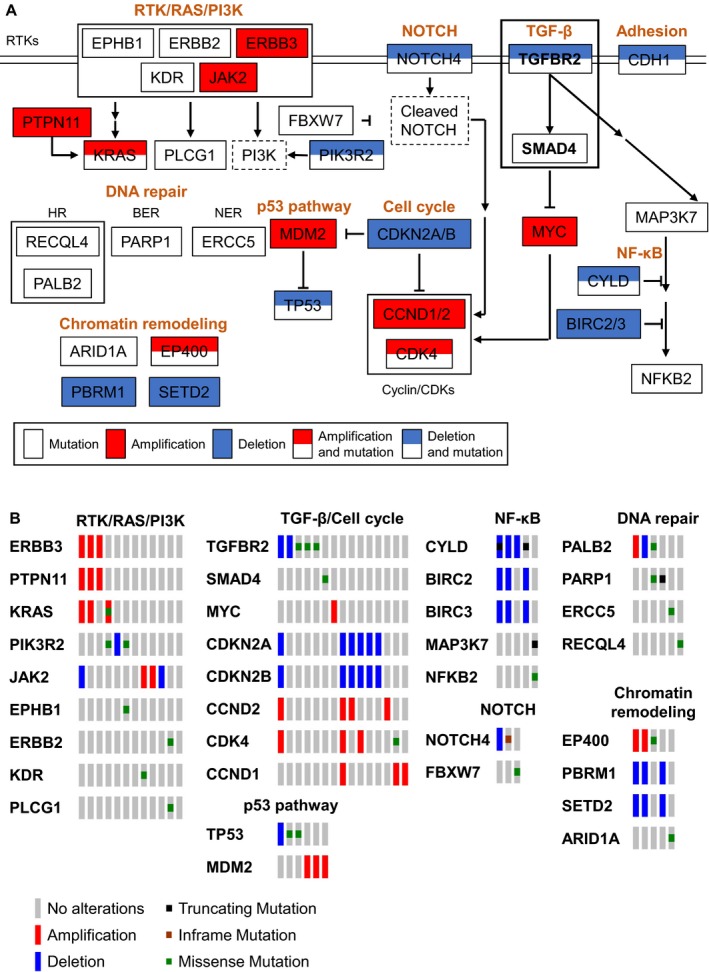
Summary pathway diagram of somatic mutations and CNVs in 33 NPC tumors. (A) Identified somatic mutations and CNVs involved in important cancer‐related pathways in this study are summarized. White, red, and blue rectangles represent point mutations, gene amplification, and deletion, respectively. Dotted rectangle represents important components involved but not altered in the pathway. (B) Somatic mutations and CNVs found in NPC tumors in cancer‐related pathways were indicated. RTK (receptor tyrosine kinase); HR (homologous recombination); BER (base excision repair); NER (nucleotide excision repair)

We observed that gene alterations in cancer‐related pathways such as RTK/RAS/PI3K, NOTCH, NF‐κB, chromatin remodeling, and DNA repair, and TGF‐β pathways. The somatic mutations and CNVs involved important cancer‐related pathways are summarized in Figure 2. Collectively, our results suggest that the identified genetic alterations in cancer pathway components, and hotspot mutations, may contribute to NPC development.

### 3.3 Copy number variation of 409 cancer panel in 33 paired NPC

Copy number variation (CNV) is a structural variation in chromosome that leads to amplification or deletion of a section of chromosome. CNVs were detected by comparing the normalized read count of a targeted gene in the tumor sample versus the nontumor tissue, as calculated using ONCOCNV.^28^ Genes located on X chromosomes were excluded from the analysis. Copy number ≥3 and ≤1 are defined as amplification and deletion, respectively. Copy number losses and gains were further validated using quantitative PCR (Figure S4). Genes found to have CNVs in three or more patients are listed in Figure 1. Interestingly, the genes identified as having copy number gains using this threshold mainly clustered at 12q13‐15, 12q24, and 12p12‐13, while genes with copy loss mainly clustered at 3p13‐14, 3p21, 9p21, 11q21‐22, 16q12, and 16q22‐23. In addition, frequent amplification of chromosomes 12 and 7q, and loss of 3p, 9p, 11q, 14q, and 16 q were identified in NPC tumors according to the numbers of gene‐level CNVs (Figure S5). A number of previously identified NPC‐driving CNVs, including amplification of the oncogenes, *KRAS* and *CCND1*, and deletion of the TSGs, *CDKN2A/2B*, and *CYLD* 12, 14 were identified in our study (Figure 1). In addition, we observed high‐level amplifications (copy number ≥ 4) among oncogenes that promote cell proliferation and cell signaling, including *MYC*, *CCND1*, and *JAK2* (Table S5).

**Figure 3:**
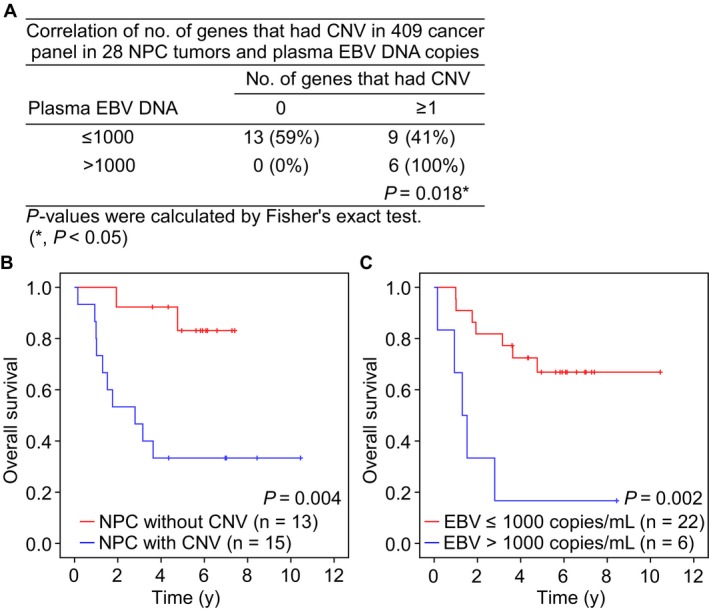
CNV events among genes in 409 cancer panel, plasma EBV DNA copies and overall survival in 28 NPC patients. (A) The correlation of CNV events with ≥1 and plasma EBV DNA copies with >1000 copies/mL were analyzed by Fisher's exact test. Kaplan‐Meier plots showing overall survival of NPC patients with (B) CNV events among the genes in 409 cancer panel and (C) EBV DNA copies in plasma. The *P*‐value of Kaplan‐Meier plots were performed using Log‐rank test

Given that EBV infection can induce genomic instability,^29^, ^30^ we examined the possible correlation between CNV events and the plasma EBV DNA concentration. We noted that plasma samples whose EBV DNA concentrations were greater than 1000 copies/mL had at least one gene (*P* = .018) in 409 cancer panel that had either gain or loss in CNV (Figure 3A), indicating possible link between plasma EBV copy number and CNV in NPC tumor.

The plasma EBV DNA concentration is considered to be a prognostic factor that can predict the treatment response and overall survival. To test whether CNVs in NPC tumors could be used as a prognostic marker, we assayed the relationship between CNV events and clinical outcome (five patients were excluded who lacked overall survival or EBV copy number information). Indeed, NPC patients who had at least one gene experiencing CNV in the 409 cancer panel tended to have poorer overall survival (*P* = .004) (Figure 3B). In addition, plasma EBV DNA concentrations greater than 1000 copies/mL were also correlated with poor overall survival (*P* = .002) (Figure 3C). These suggest that the occurrence of at least one CNV among the genes in 409 cancer panel and high plasma EBV DNA concentrations might be used as indicators to predict poor survival outcome in NPC patients.

### 3.4 Mutations in TGFBR2 and SMAD4 interfere the TGF‐β/SMAD activation

In an effort to use the gene alterations discovered in the 409 cancer‐related genes panel to gain insight into NPC pathogenesis, we performed functional analyses using the Metacore and KEGG bioinformatics resources. Consistent with previous reports,^12^, ^14^, ^15^, ^17^ the ErbB‐PI3K and NF‐κB signaling pathways were altered in NPC. In addition, TGF‐β receptor signaling (Table S6) and adherens junction pathways (including *MAP3K7*, *ERBB2*, *TGFBR2*, *SMAD4*, and *CDH1*) (Table S7) were altered. Two NPC samples harbored the same mutation within the kinase domain of TGFBR2 (G253V) (NPC_003T and NPC_009T) and a third exhibited the TGFBR2 (E290K) (NPC_048T) mutation. Neither mutation has been previously reported in NPC. In addition, one sample (NPC_076T) was found to harbor the SMAD4 R361H mutation, which is a known hotspot mutation that interferes TGF‐β‐mediated signaling.^25^ Thus, we further studied mutations involved in the TGF‐β signaling pathway (*TGFBR2* and *SMAD4*) and evaluated their phenotypes in the context of NPC tumorigenesis.

**Figure 4:**
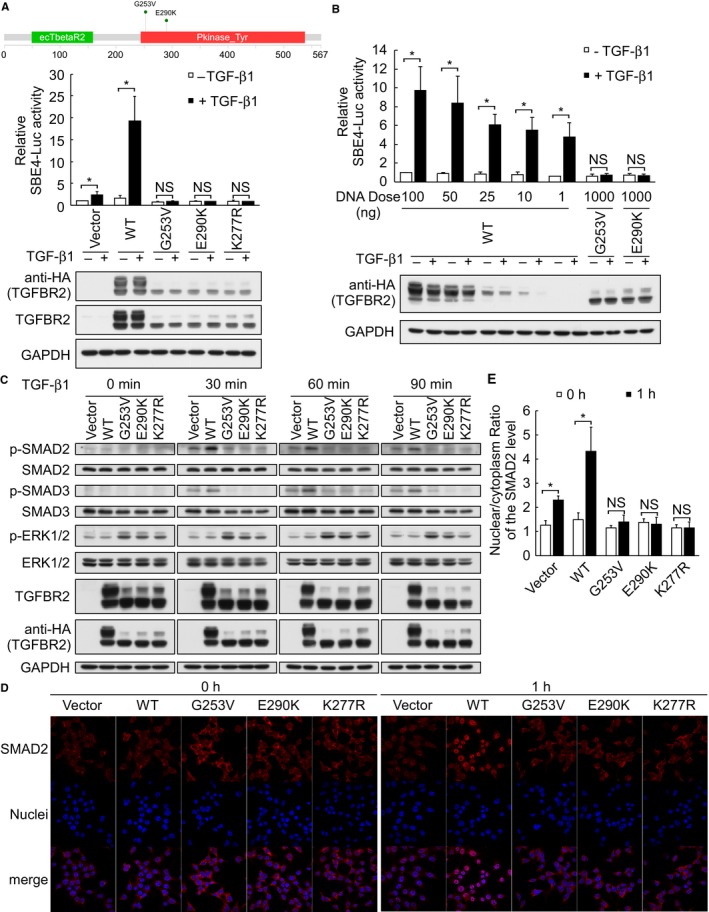
TGFBR2 G253V and E290K loss‐of‐function mutations inactivate TGF‐β/SMAD signaling. (A) Upper panel represents the schematic diagram of the TGFBR2 mutations identified in this study, as generated by the MutationMapper visualization tool. (A) and (B) HEK293T cells were cotransfected with constructs encoding HA‐tagged WT or mutant TGFBR2, SBE4‐Luc, and pRL‐TK, and replaced with serum‐free DMEM after 6 h. Cells were maintained in the serum‐free medium for another 18 h before stimulated with or without 5 ng/mL TGF‐β1 for 15 h. Promoter activities were measured using a dual luciferase assay system. Firefly luciferase activities were normalized to those of Renilla luciferase. (C) Immunoblot analysis of TGF‐β/SMAD‐ and non‐SMAD signal transduction‐related components in HEK293T cells transfected with empty vector or vectors encoding WT or mutant TGFBR2 for 24 h and then stimulated with 5 ng/mL TGF‐β1 for 0, 30, 60, or 90 min. (D) and (E) Immunofluorescence analysis of SMAD2 translocation in HEK293T cells transfected with empty vector or vectors encoding HA‐tagged WT or mutant TGFBR2 for 24 h and then stimulated with 5 ng/mL TGF‐β1 for 0 or 1 h. The nuclear/cytoplasmic ratios of SMAD2 were quantified using an IN Cell Analyzer. Values on bar graphs were shown as the mean ± SD of three independent experiments with duplicates. Intergroup comparisons were conducted by using Student's *t* test. (NS, not significant; *P < 0.05)

It is well known that upon TGF‐β stimulation, both SMAD2 and SMAD3 are phosphorylated to form a complex with SMAD4. This complex is translocated into the nucleus to activate target genes containing SMAD4 binding sites. To examine whether the identified mutations in *TGFBR2* might be relevant to TGF‐β/SMAD signaling, we cotransfected cells with the TGF‐β signaling effector luciferase reporter, SBE4‐Luc (containing four copies of the SMAD‐binding site, GTCTAGAC), plus empty vector or various hemagglutinin (HA)‐tagged TGFBR2 expression plasmids encoding the wild‐type (WT) protein, the two identified TGFBR2 mutants, or the kinase‐dead K277R mutant (as a negative control). The transfected cells were treated with or without TGF‐β and reporter gene expression was assessed. As shown in Figure 4A, WT TGFBR2 could activate the luciferase reporter containing the TGF‐β effector, whereas the two identified TGFBR2 mutants (G253V and E290K) and the kinase‐dead mutant were incapable of transmitting the TGF‐β signal. To test whether the SMAD4 (R361H) mutant is responsive to TGF‐β, we cotransfected MBA‐MD‐468 cells with the SBE4‐Luc reporter plus empty vector or flag‐tagged expression plasmids encoding WT or mutant SMAD4, and treated the cells with or without TGF‐β. As expected, WT SMAD4 activated the SBE4‐Luc reporter and responded to TGF‐β, whereas SMAD4 (R361H) did not (Figure S6). To examine whether these TGFBR2 mutants could disrupt the SMAD and/or non‐SMAD TGF‐β signaling pathways, we transfected HEK293T cells with empty vector or HA‐tagged WT or mutant TGFBR2 expression plasmids and used Western blot analysis to detect phosphorylation of SMAD2 and SMAD3, as indicators of signaling pathway activation, upon TGF‐β stimulation. As shown in Figure 4C, the three TGFBR2 mutants failed to induce phosphorylation of SMAD2 or SMAD3 at 30, 60, or 90 min after the induction of TGF‐β stimulation, whereas such phosphorylation was seen in the vector and WT groups. In contrast, expression of the three TGFBR2 mutants increased the basal level of extracellular signal‐regulated kinases (ERK) 1/2 phosphorylation (a marker of the non‐SMAD TGF‐β pathway), regardless of TGF‐β stimulation (Figure 4C). Consistent with the results of our Western blot analysis, immunofluorescence imaging showed that HEK293T cells transfected with the two NPC‐related TGFBR2 mutants exhibited reduced translocation of SMAD2 compared to WT‐transfected cells at 60 min of TGF‐β treatment (Figure 4D,E). This indicates that the NPC‐related TGFBR2 kinase domain mutations blocked TGF‐β signal transduction by abrogating the proper phosphorylation of SMAD. Although consistent amounts of DNA were transfected into cells, the protein levels of the TGFBR2 mutants were lower than those of the exogenous WT protein (Figure 4A). The underlying reason for this is currently unknown. To test whether this change in protein expression accounted for the observed inhibition of TGF‐β‐mediated activation in cells expressing the TGFBR2 mutants, we transfected HEK293T cells with 100, 50, 25, 10, and 1 ng of the WT TGFBR2 plasmid and used Western blot analysis to determine the WT protein expression level. As shown in Figure 4B, cells transfected with 10‐25 ng of WT TGFBR2 plasmid expressed the WT protein at levels that were comparable to those seen in cells expressing the mutant proteins, and this level of WT protein could still activate TGF‐β signaling. Together, our results suggest that the TGFBR2 (G253V) and TGFBR2 (E290K) mutants yield phenotypes similar to those associated with the previously reported kinase‐dead mutant, K277R, all three of which failed to transduce TGF‐β/SMAD downstream signals through the phosphorylation and subsequent nuclear translocation of SMAD2.

### 3.5 TGFBR2 mutant‐expressing cells fail to show TGF‐β‐mediated growth inhibition

**Figure 5:**
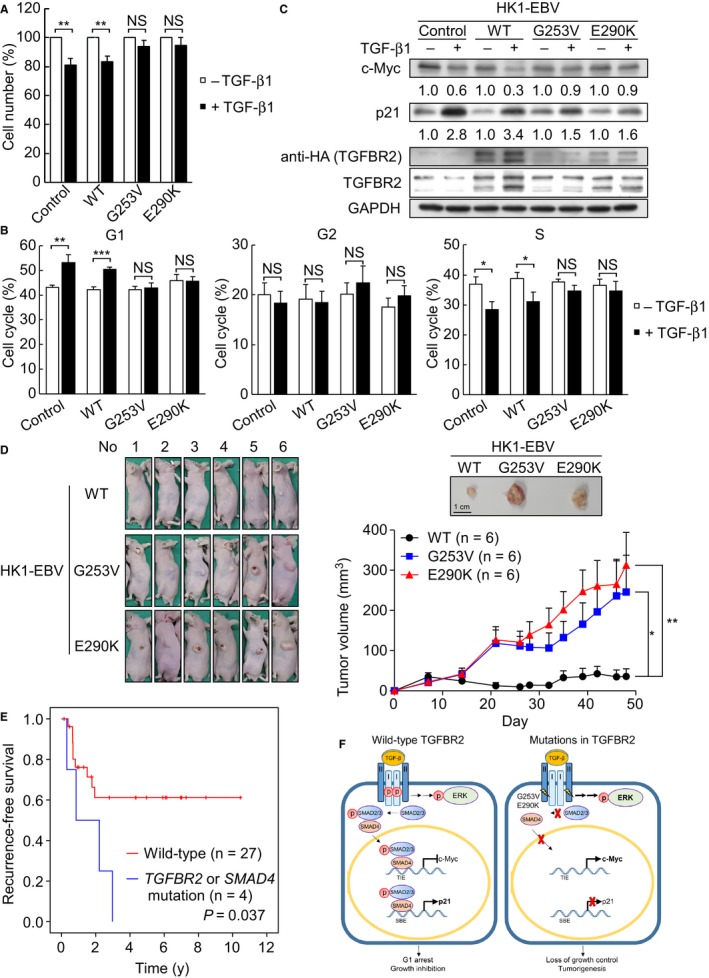
TGFBR2 loss‐of‐function mutations are resistant to TGF‐β‐induced growth inhibition and predicted poorer recurrence‐free survival of 31 NPC patients. (A) Control HK1‐EBV cells and those expressing HA‐tagged WT or mutant TGFBR2 were cultured with or without 5 ng/mL TGF‐β1 for 48 h. Cell number was determined by the CCK‐8 assay. (B) Control HK1‐EBV cells and those expressing WT or mutant TGFBR2 were cultured with or without 5 ng/mL TGF‐β1 for 48 h and the cell cycle was analyzed by flow cytometry. (C) Western blot analysis of the indicated proteins after 48 h of TGF‐β stimulation in control HK1‐EBV cells and those expressing WT or mutant TGFBR2. (D) Loss of growth inhibition by TGFBR2 mutants in vivo. Tumor growth of TGFBR2‐expressing HK1‐EBV cells in a xenograft model. Photographs showed the appearance of tumor‐bearing nude mice and representative tumor gross. Tumor volumes were measured at day 7, 14, 21, 26, 28, 32, 35, 39, 42, 46, and 48 postinoculation, respectively. Data represented as the mean ± SEM (n = 6) and were analyzed by Student's *t* test. (E) Kaplan‐Meier recurrence‐free survival curves for NPC patients with mutations in TGF‐β/SMAD signaling pathway (*P* = .037; Log‐rank test on 31 NPC cases). (F) The role of TGFBR2 G253V and E290K loss‐of‐function mutations in TGF‐β/SMAD signaling pathway. Cells expressing WT TGFBR2 remain sensitive to TGF‐β‐mediated growth inhibition by downregulating proliferating gene, c‐Myc, and activating cell arrest gene, p21, upon TGF‐β stimulation. Conversely, NPC cells expressing mutant versions of TGFBR2 activate ERK phosphorylation but fail to transmit signal along the TGF‐β/ SMAD‐dependent pathway, leading to c‐Myc activation and p21 suppression. Finally, the TGFBR2 mutations (G253V and E290K) promote uncontrollable cell growth and tumorigenesis in NPC cells. TIE (TGFβ‐inhibitory element); SBE (Smad‐binding element). Values on bar graphs were shown as mean ± SD of three independent experiments. Intergroup comparisons were conducted by using Student's *t* test. (NS, not significant; *P < 0.05; **P < 0.01; ***P < 0.001)

TGF‐β potently inhibits cell growth by inducing G1 cell cycle arrest. To determine whether the identified TGFBR2 mutants evade TGF‐β‐mediated growth inhibition, we established HK1‐EBV clones that stably expressed WT or mutant TGFBR2. Addition of TGF‐β inhibited the growth of control and WT *TGFBR2* cells, but failed to inhibit the cell growth of the two stable TGFBR2 mutant cell lines (Figure 5A). Subsequent flow cytometric analysis of the cell cycle content revealed that TGF‐β could induce G1 arrest and reduce proliferation and S phase in the control and WT cell lines but not in the two mutant cell lines (Figure 5B). Furthermore, upon TGF‐β treatment, Western blot analysis of TGF‐β target genes involved in the cell cycle showed that the proliferation marker, c‐Myc, was downregulated to 0.6‐ and 0.3‐fold in control and WT cells, respectively; whereas the G1 arrest marker, p21, was significantly upregulated to 2.8‐ and 3.4‐fold in control and WT cells, respectively (Figure 5C). In contrast, in TGFBR2 mutants upon TGF‐β treatment, the protein levels of c‐Myc were similar with or without TGF‐β treatment; whereas the protein levels of p21 were slightly upregulated to 1.5‐ and 1.6‐fold (Figure 5C). These suggest that cells expressing the NPC‐associated TGFBR2 mutants were less sensitive to TGF‐β‐induced growth inhibition and thus would be prone to uncontrolled proliferation.

We evaluated the effect of TGFBR2 mutants on tumor growth in vivo by inoculating HK1‐EBV‐expressing WT and TGFBR2 mutants in nude mice. The tumor volumes of HK1‐EBV‐expressing WT TGFBR2 were significantly smaller than those of TGFBR2 mutants from day 21 (Figure 5D, *P* < .05). At the end of the experiment (day 48), the average of tumor volumes of TGFBR2 mutants, G253V and E290K, was 6.9‐ and 8.8‐fold, respectively, larger than that of WT TGFBR2, indicating that TGFBR2 mutants promote tumor growth (Figure 5D).

Given our finding that cells expressing both TGFBR2 mutants had defects in SMAD‐related TGF‐β‐responsive signaling but not in non‐SMAD TGF‐β‐responsive signaling (Figure 4), we assessed whether the identified genetic changes in *TGFBR2* and *SMAD4* were associated with clinical outcome. Two patients who lacked recurrence survival information were excluded. In the studied set of 31 primary NPC tumor biopsies, 13 of the patients had local recurrence while the other 18 experienced complete remission. Indeed, Kaplan‐Meier survival analysis showed that NPC patients with mutations in *TGFBR2* and *SMAD4* tended to have poor local recurrence‐free survival (*P* = .037) as compared to patients without *TGFBR2* and *SMAD4* mutations (Figure 5E).

## 4 DISCUSSION

The identification of genetic alterations via comprehensive sequencing of tumor genomes has proven to be a powerful and important tool for cancer diagnosis. However, whole‐genome and WES remain cost‐prohibitive for routine use. The targeted sequencing of the genes contained within comprehensive high‐depth cancer panels may offer a cost‐effective approach for identifying cancer‐related mutations and potential actionable targets. High‐depth sequencing may also accurately reveal low‐frequency oncogenic driver mutations in low‐purity, heterogeneous tumor samples. Importantly, the CCPs typically include most of the potential druggable targets, making such work beneficial for patients.

Mutations in *TP53*, *ARID1A*, *EP400*, and *SYNE1* are detected in this study and other NPC WES studies^12^, ^14^, ^15^ (Figure 1), suggesting that these gene mutations are common in NPC. Notably, our present study identified mutations in components of signaling pathways previously found to be enriched in NPC WES studies, including the PI3K/MAPK, NOTCH, NF‐κB, chromatin remodeling, and DNA repair pathways.^12^, ^14^, ^15^, ^17^ For example, the deubiquitinase, CYLD, which is a negative regulator of the NF‐κB pathway, was found to have undergone inactivating mutations (I903fs and S371X leading to protein truncation due to stop‐gain or frameshift) in NPC patients of two Hong Kong‐based studies.^14^, ^15^ Based on the present and previous findings, we suggest that the absence of the negative regulatory function of CYLD allows the oncogenic NF‐κB pathway to become overactivated, and that this may contribute to NPC development. Due to the limited number of genes covered in CCP, it is likely other NF‐κB‐related genes reported in NPC have not examined in this study.^14^, ^15^

We identified a number of oncogenes and TSGs that exhibited CNVs (both gains and losses) in NPC (Figure 1). These genes could prove useful as drug targets and molecular biomarkers for the presence and/or prognosis of NPC. Previously, a WES‐based study reported arm‐level CNVs in NPC.^14^ This would affect many genes, including potential oncogenic driver genes and TSGs. Due to the limitation of 409 cancer‐related genes panel, a number of genes that might have CNVs were underestimated in the present study. We found that *ZNF384* and *ING4* (12p13.31, 5/33, 15%) were frequently amplified in NPC, while *SETD2* (3p21.31, 3/33, 9%), *BIRC2*, and *BIRC3* (11q22.2, 3/33, 9%) were frequently deleted (Figure 1). Similar results were also reported in other studies, for instance, the 12p13 region was previously reported to be highly amplified in NPC, as assessed in a comparative genomic hybridization study 31 and regions of 3p21.31 and 11q22.2 were reported to be critical for tumor suppression in NPC.^32^, ^33^, ^34^

Our pathway analysis revealed that multiple components of TGF‐β signaling may be altered in NPC. Several inactivating TGFBR2 mutations have been reported in various cancers, including truncation mutations in microsatellite‐instable patients as well as missense mutations in the kinase domain.^35^ One study reported a TGFBR2 mutation that enhanced TGF‐β signaling.^36^ Here, we report two new TGFBR2 missense mutations that inhibit SMAD‐dependent TGF‐β signaling (Figure 4). These mutations are both located in the kinase domain, close to the adenosine triphosphate (ATP)‐binding pocket, and thus may affect ATP binding to inactivate TGFBR2 kinase activity. Sequence analysis showed that the residues altered by the two mutations are conserved among the members of the TGF‐β receptor family, and thus may be functionally and evolutionarily important.^37^ Downregulation of TGF‐β signaling by miR‐93‐targeting TGFBR2^38^ and by TNFRSF19 interaction with TGF‐β receptor I^39^ has been reported in NPC to counteract growth inhibition mediated by TGF‐β. In agreement with these previous findings, we showed that these two mutations in TGFBR2 also prevented the growth inhibition.

The total expression of mutant TGFBR2 was lower than that of the WT protein in both transiently transfected HEK293T cells and stable HK1‐EBV cell lines (Figures 4A‐C and 5C). It is possible that the mutations in the ATP‐binding pocket may cause a conformational change that leads to protein instability. We also observed less of the higher molecular weight protein (ie, the glycosylated form; >72 kDa) in HEK293T cells transiently expressing the TGFBR2 mutants versus the WT protein (Figure 4A‐C). Future work is needed to investigate whether these TGFBR2 mutations affect the trafficking and/or glycosylation of the protein, and whether receptor glycosylation affects the protein's stability and ability to transduce canonical TGF‐β signaling.

In addition to the canonical SMAD‐dependent pathway, TGF‐β can also induce non‐SMAD pathways, including those mediated by ERK, JNK, p38 MAPK, IKK, PI3K‐AKT, and the Rho family GTPases.^40^ Increasing evidence demonstrates that non‐SMAD ERK signaling is associated with TGF‐β1 signaling in cancer progression.^41^ The details of the TGF‐β/ERK1/2 signaling pathways differ by cell types and tissues. Inactivation of ERK abrogates TGF‐β‐mediated tumor‐promoting effects but does not alter TGF‐β‐mediated tumor suppression.^42^ Here, the identified TGFBR2 mutants appear to play an oncogenic role by switching off tumor‐suppressive TGF‐β‐stimulated SMAD signaling while leaving TGF‐β‐stimulated ERK signaling intact (Figure 4C). Thus, these mutants lose their tumor‐suppressive function while gaining oncogenic potential. Together, the previous and present results suggest that the SMAD and non‐SMAD pathways play distinct role for TGFBR2.

Overall, our results support a model (Figure 5F) in which cells expressing the two TGFBR2 mutants fail to undergo TGF‐β‐stimulated SMAD2/3 signaling responses, including c‐Myc suppression and p21 activation, but do not show any alteration in the non‐SMAD ERK pathway. The subsequent activation of c‐Myc but inactivation of p21 could stimulate growth and NPC tumorigenesis. NPC patients with TGF‐β signaling mutations tend to have poor survival and more aggressive cancerous outcomes, and thus may require more frequent posttreatment follow‐up care and effective interventions.

## CONFLICT OF INTEREST

The authors declare no conflict of interest.
