# Supplementary figures and images for: Targeted sequencing of cancer‐related genes in nasopharyngeal carcinoma identifies mutations in the TGF‐β pathway
Source: Cancer Med. 2019 Jul 22;8(11):5116–27. doi: 10.1002/cam4.2429 (PMC6718742; doi:10.1002/cam4.2429)

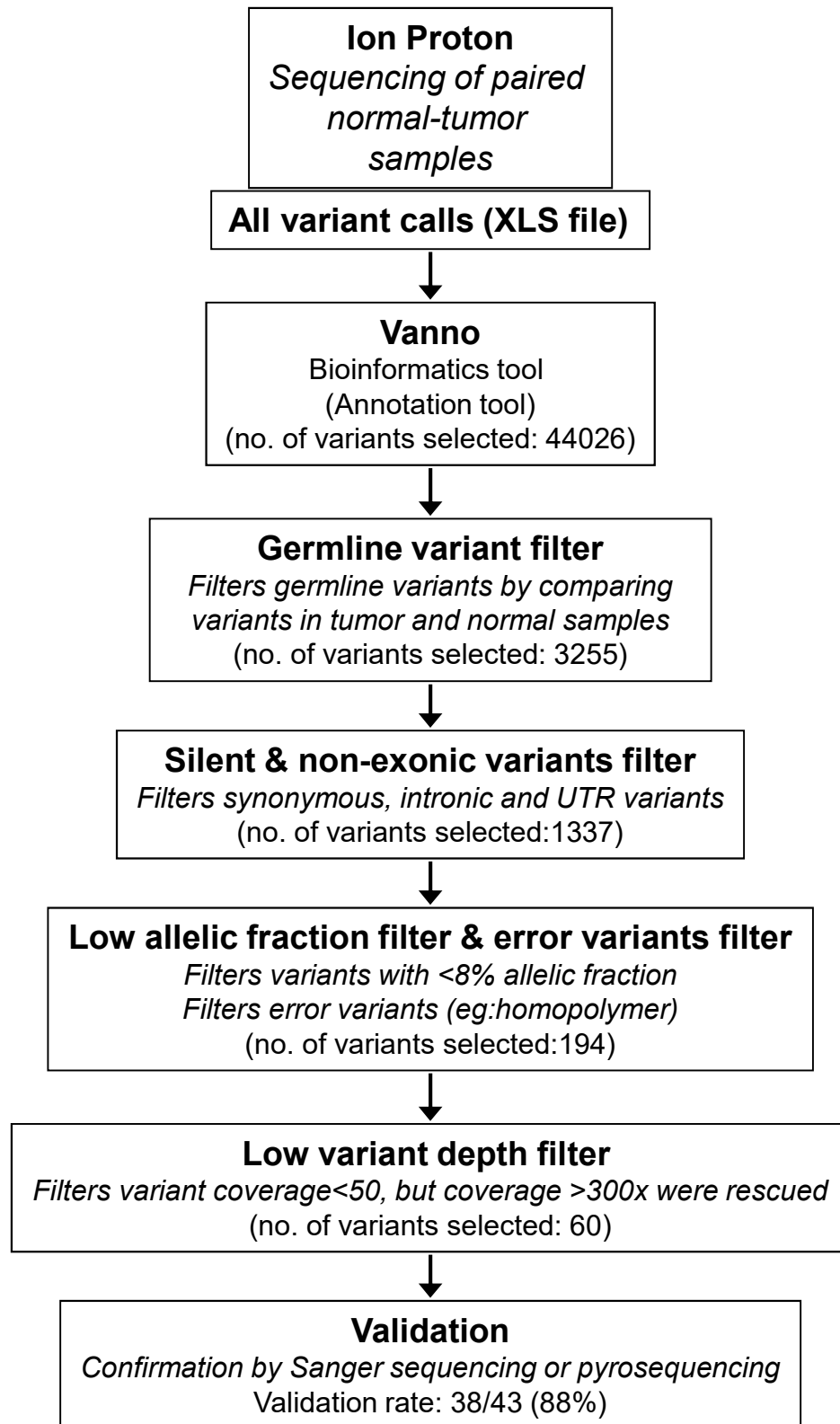

**Fig. S1. Flow chart showing the step-wise analysis of our next-generation sequencing data.**

Supplement: Supplementary file 1 [file CAM4-8-5116-s001.pdf]

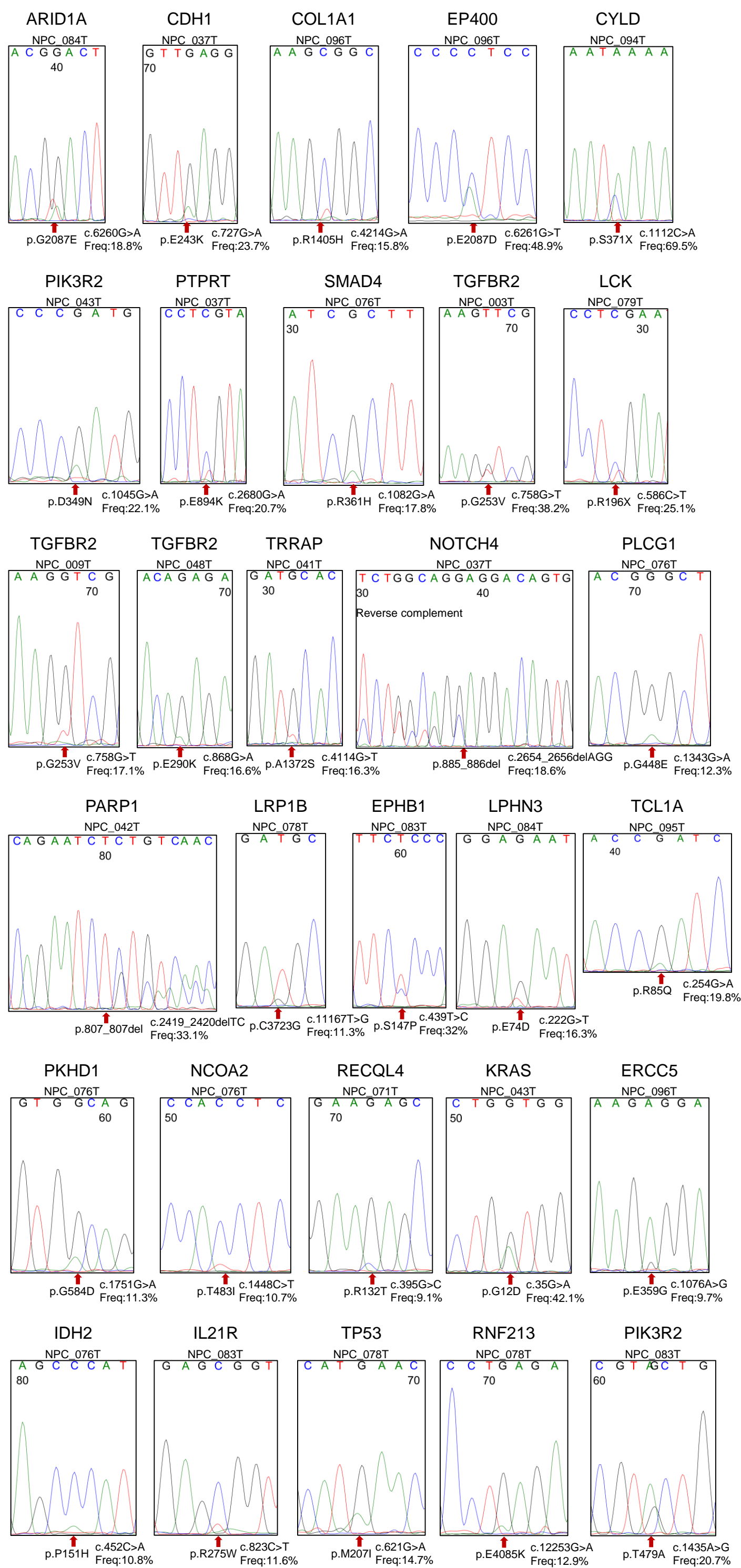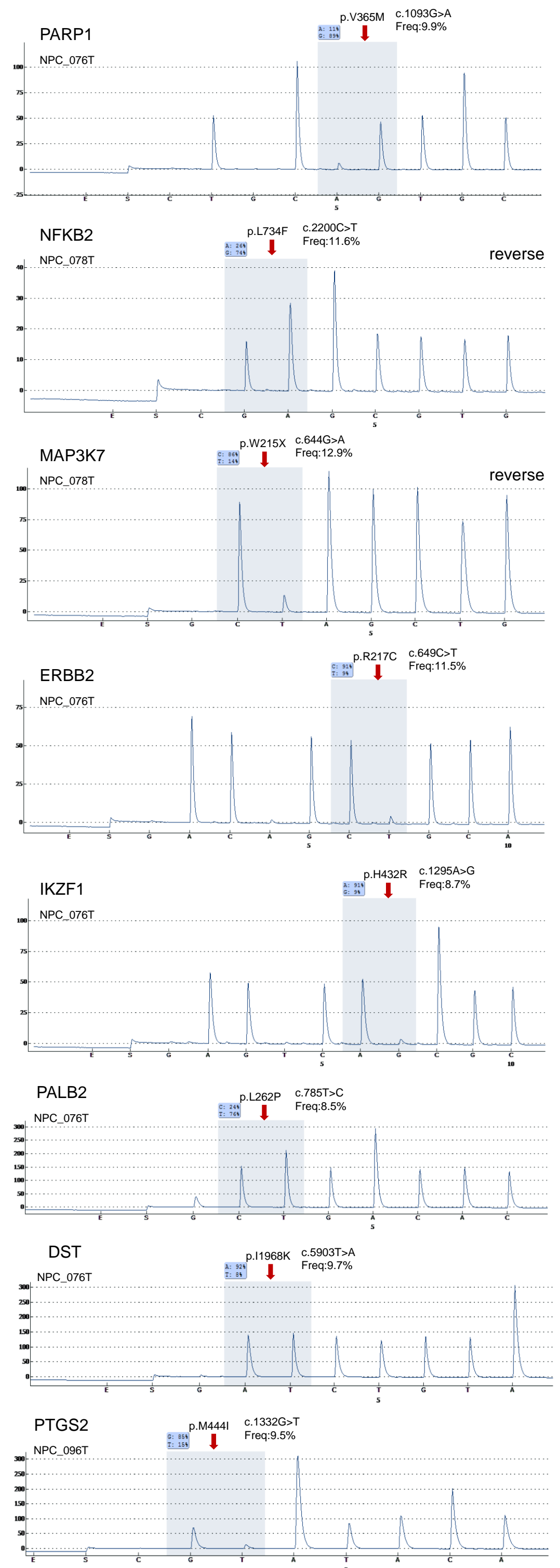

**Fig. S3. Validation of select mutations by Sanger sequencing or pyrosequencing**

Supplement: Supplementary file 3 [file CAM4-8-5116-s003.pdf]

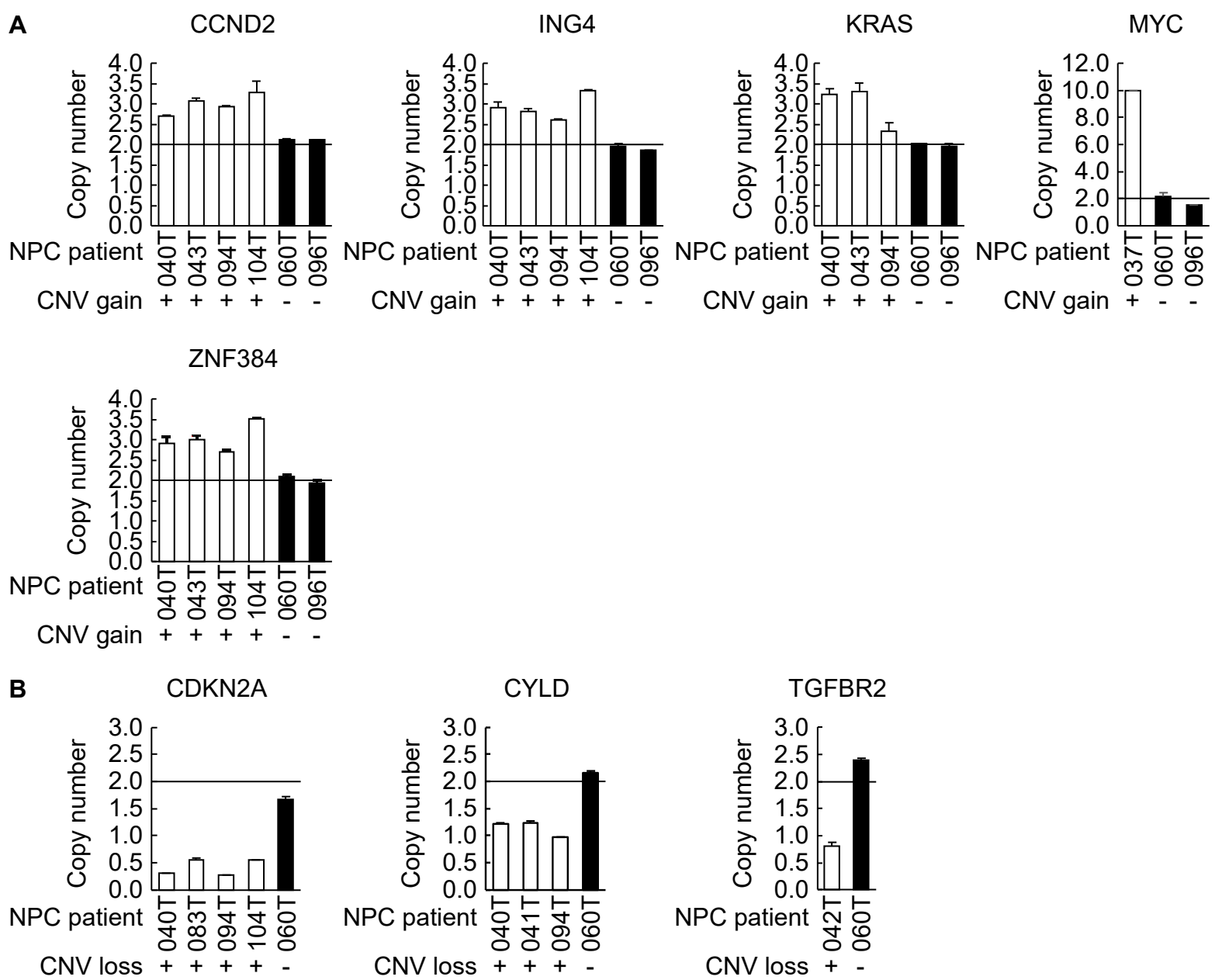

**Fig. S4. Validation of copy number variations (CNVs) by quantitative real-time PCR.**

Supplement: Supplementary file 4 [file CAM4-8-5116-s004.pdf]

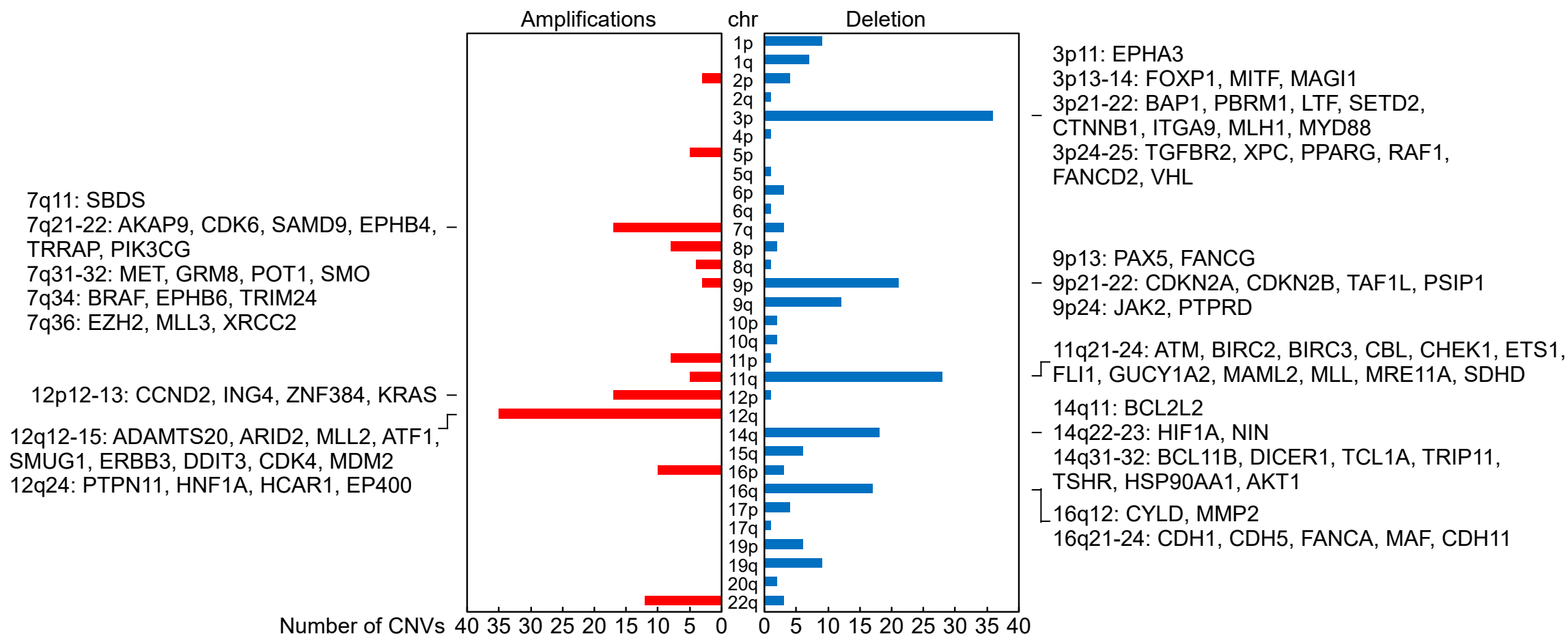

**Fig. S5. Number of genes with CNVs in 33 NPC patients were counted according to the chromosome region.**

Supplement: Supplementary file 5 [file CAM4-8-5116-s005.pdf]
