# Supplementary material for: Targeted sequencing of cancer‐related genes in nasopharyngeal carcinoma identifies mutations in the TGF‐β pathway
Source: Cancer Med. 2019 Jul 22;8(11):5116–27. doi: 10.1002/cam4.2429 (PMC6718742; doi:10.1002/cam4.2429)
Supplement: Supplementary file 2 [file CAM4-8-5116-s002.pdf]

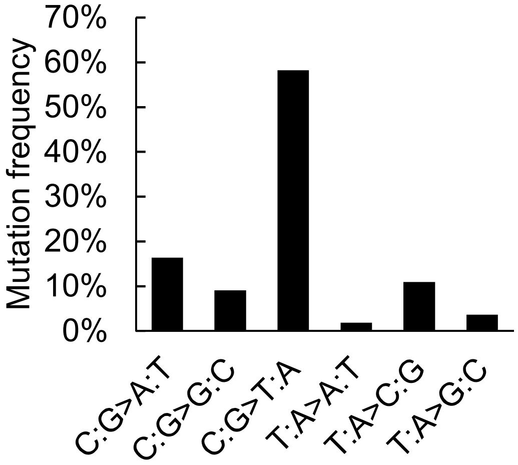

Fig. S2. The frequencies of the six classes of base substitutions generated by somatic mutations in NPC.
