# Supplementary material for: Targeted sequencing of cancer‐related genes in nasopharyngeal carcinoma identifies mutations in the TGF‐β pathway
Source: Cancer Med. 2019 Jul 22;8(11):5116–27. doi: 10.1002/cam4.2429 (PMC6718742; doi:10.1002/cam4.2429)
Supplement: Supplementary file 6 [file CAM4-8-5116-s006.pdf]

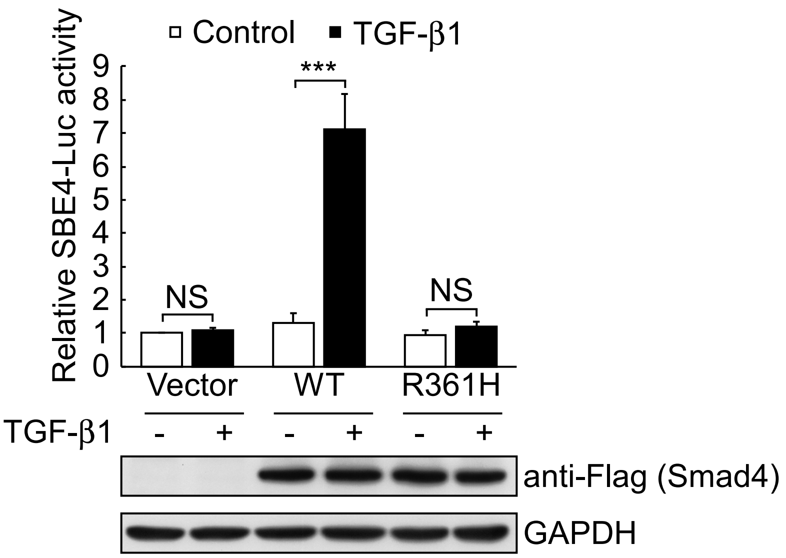

Fig. S6. The R361H loss-of-function mutation in SMAD4 inactivatesTGF-β/SMAD signaling in MBA-MD-468 cells.
