# Supplementary material for: Targeted sequencing of cancer‐related genes in nasopharyngeal carcinoma identifies mutations in the TGF‐β pathway
Source: Cancer Med. 2019 Jul 22;8(11):5116–27. doi: 10.1002/cam4.2429 (PMC6718742; doi:10.1002/cam4.2429)
Supplement: Supplementary file 14 [file CAM4-8-5116-s014.docx]

**Doc S1.** Supporting information**.** Materials and methods, and supplementary figure legends were shown in this document.

**Materials and Methods**

**Specimens and DNA extraction**

A total of 33 freshly frozen primary biopsies of tumor samples and matched control samples (28 PBMC samples and 5 adjacent normal tissues) from NPC patients admitted to Chang Gung Memorial Hospital at Linkou during 2003 to 2011 (Table S2). All samples were approved by the institutional review board and ethics committee of Chang Gung Memorial Hospital, Linkou, Taiwan (IRB number, 98-2552B). Genomic DNA was extracted using a QIAamp DNA Mini kit (Qiagen).

**Next generation sequencing and analysis**

Sequencing libraries for each sample were prepared using an Ion AmpliSeq Comprehensive Cancer Panel primers (Life Technologies) according to the manufacturer’s instructions. Emulsion PCR (emPCR) was carried out by amplification of template on Ion sphere particles (ISPs) using the Ion OneTouch System (Life Technologies) following the manufacturer’s instructions. The enriched template positive ISPs were loaded and sequencing on an Ion PI™ Chip v2 according to the Ion PI™ Sequencing 200 Kit v2 protocol (Life Technologies).

The sequencing data were processed using the Ion Torrent Suite software (v4.4; Life Technologies), which aligned each obtained sequence to the targeted regions, calculated the coverage, and called variants using the default somatic low-stringency settings. The variants were annotated and predicted as deleterious mutation by six algorithms (SIFT, PolyPhen2, LRT, MutationTaster, MutationAssessor and FATHMM) using Vanno ^1^. To filter out germline variants, the sequences obtained from tumors and matched normal samples were compared. Sequencing errors as well as silent and non-exonic variants were filtered out. Mutations with allele frequencies ≥ 8% to reduce false-positive were selected. Variant depth threshold was set at ≥50 x to avoid errors; however, variants with total coverages ≥300 x were rescued.

CNV was analyzed by applying sequencing data to ONCOCNV ^2^. The q-value cutoff was set at 0.1. CNVs were generated both in a paired manner and by normalization of read coverage with a pool of normal control samples. The results were divided into four groups: >4 copies, <4 to ≥3 copies, <3 to >1 copies, and ≤1 to 0 copies. CNVs generated from both methods fell into the same group, they were considered to be confident. Genes with copy number variations <1.5 or >2.5 were considered as copy number losses or gains, respectively.

**Validation of mutations by Sanger sequencing and pyrosequencing**

Mutations were verified by Sanger sequencing or pyrosequencing. Sequencing primers were listed in the Table S3.

**Validation of CNVs by quantitative real-time PCR**

The CNVs were verified by quantitative real-time PCR using the FastStart DNA Master SYBR Green I reagent (Roche Diagnostics). *HSP90AB1* was assessed as a control gene and was considered as two copies per cell. Human leukocyte DNA from healthy donors was used to generate a standard curve to determine the amount of each amplicon in the tumor samples. The CNVs were calculated by comparing the Ct value of the target gene in the tumor sample versus that in the human leukocyte DNA, and then normalized to the control gene. The primers used in the reactions were listed in Table S3.

**Plasmids**

Plasmids obtained from Addgene: SBE4-Luc was a gift from Bert Vogelstein (Addgene plasmid # 16495); pCMV5B-TGFbeta receptor II wt was a gift from Joan Massague & Jeff Wrana (Addgene plasmid # 11766); pcDNA Flag-Smad4M was a gift from Joan Massague (Addgene plasmid # 14959). Plasmids encoding the HA-tagged TGFBR2 mutants (G253V and E290K) were generated by site-directed mutagenesis using either pCMV5B-TGFbeta receptor II wt (for DNA transfection) or pLKO-AS2.puro-TGFBR2-HA (for lentivirus infection).

**Immunoblot analysis**

Immunoblot analysis assay was carried out on SDS-PAGE, and proteins were transferred to membranes. Anti-p-ERK1/2 (#9101), anti-SMAD2 (#5339), anti-p-SMAD2 (#3108), anti-SMAD3 (#9523), and anti-p-SMAD3 (#9520) antibodies were purchased from Cell Signaling. Anti-ERK1/2 (sc-93), anti-GAPDH (sc-32233) anti-HA (sc-7392), anti-c-Myc (sc-40), anti-p21 (sc-6246) and anti-TGFBR2 (sc-220) antibodies were purchased from Santa Cruz.

**Luciferase assay**

HEK293T cells and MDA-MB468 cells, a SMAD4-null cell line, were used to analyze WT and mutant, TGFBR2 and SMAD4 plasmids, respectively, in response to TGF-β. HEK293T and MDA-MB468 cells were co-transfected with 1 μg of SBE4-Luc, 10 ng of pRL-TK (Promega) and 1 μg of constructs encoding, TGFBR2 and SMAD4, respectively, and washed with serum-free medium. After 24 h, cells were stimulated with or without 5 ng/ml TGF-β1 (Peprotech) for 15 h. Cells were lysed and promoter activity was measured using a dual luciferase assay system (Promega). Firefly luciferase activities were normalized with respect to that of Renilla luciferase.

**Immunofluorescence staining**

HEK293T cells on glass coverslips were transfected with 2 μg of empty vector or vectors encoding WT or mutant TGFBR2. After 24 h, cells were washed three times with serum-free medium. Cells were stimulated with or without 5 ng/ml TGF-β1 (Peprotech) for 1 h. Cells were washed, fixed with 3.7% formaldehyde, permeabilized with 0.1% Triton X-100, incubated with anti-SMAD2 antibody, and incubated with an Alexa Fluor 488-conjugated secondary antibody. Nuclei were stained with DAPI and the results were examined by confocal microscopy. The nuclear/cytoplasmic ratio of the SMAD2 level was quantified using GE Healthcare Investigator (GE Healthcare).

**Lentiviral production and transduction**

HEK293T cells were used for lentivirus packaging and HK1-EBV cells were used for lentivirus infection experiments. Lentiviral vector pLKO-AS2.puro was purchased from National RNAi Core Facility Platform (RNAi Core Facility Platform, Academia Sinica). HA-tagged WT or mutant TGFBR2 were cloned into the pLKO-AS2.puro expression vector. HEK293T cells were co-transfected with lentivirus packaging plasmids: pMD.G, pCMVΔR8.91 and pLKO-AS2.puro expression vector (RNAi Core Facility Platform, Academia Sinica). After 6 h, transfection medium was replaced with DMEM supplemented with 1% FCS. Lentivirus-containing supernatants were harvested at 48 h after transfection and used to infect HK1-EBV cells overnight in the presence of 8 µg/mL polybrene. The transduced NPC cells were selected with 1 μg/ml puromycin.

**Cell proliferation assay**

Lentivirus-infected HK1-EBV cells (1 × 10^4^ cells/well) were seeded to 48-well plates and incubated overnight. Cells were treated with 5 ng/ml TGF-β1 (Peprotech) for 48 h, and cell number was determined using the CCK-8 assay (Dojindo).

**Flow cytometric analysis of cell cycle**

Cells were fixed overnight with 70% ethanol, incubated with 100 μg/ml RNase A for 30 min at 37°C, and stained with 10 ng/ml propidium iodide (PI) (Cat # P4170, Sigma-Aldrich). The cell cycle distributions were determined by PI fluorescence using flow cytometry.

**Animal tumor models**

Mouse experiments were approved by the Institutional Animal Care and User Committee of Chang-Gung University. Nude mice (6-week-old male) were purchased from National Laboratory Animal Center, Taiwan and BioLASCO, Taiwan. The tumors were established by subcutaneous injection with 2 × 10^6^ tumor cells. Tumor volume were determined once (day 0 to 14) or twice (day 15 to 48) weekly.

**Statistical analysis**

Statistical analyses were performed using the SPSS 13.0 software. Survival times between groups was assessed using Kaplan–Meier method and log-rank test. Categorical data was analyzed with Fisher's exact test. Experimental data were calculated using two-tailed Student’s t test. *p*-values < 0.05 were considered statistically significant.

**References**

[1] Huang PJ, Lee CC, Tan BC, et al. Vanno: a visualization-aided variant annotation tool. Hum Mutat. 2015; 36: 167-74.

[2] Boeva V, Popova T, Lienard M, et al. Multi-factor data normalization enables the detection of copy number aberrations in amplicon sequencing data. Bioinformatics. 2014; 30: 3443-50.

**Supplementary Figure legends:**

**Fig. S1.** Flow chart showing the step-wise analysis of our next-generation sequencing data.

**Fig. S2.** The frequencies of the six classes of base substitutions generated by somatic mutations in NPC.

**Fig. S3.** Validation of select mutations by Sanger sequencing or pyrosequencing. Thirty-eight of the identified variants (2 indels and 36 point mutations) were verified by (A) Sanger sequencing or (B) pyrosequencing. Red arrows indicate the mutation sites. The cDNA coordinates and nucleotide changes are shown beside each arrow.

**Fig. S4.** Validation of CNVs by quantitative real-time PCR. The (A) amplifications and (B) deletions identified by NGS were validated by SYBR Green qRT-PCR. HSP90AB1, which is located on 6p21.1 within an infrequent copy number alteration region in NPC, was used as a control gene. CNVs were calculated by comparing the Ct values obtained for each target gene in a tumor sample with that in human leukocyte DNA, and then normalized to the control gene. Two DNA samples from healthy donors were used as controls in each qPCR reaction. Data are shown as mean ± SD with duplicate.

**Fig. S5.** Analysis of CNV events on chromosomal regions. Number of genes with CNVs in 33 NPC patients were counted according to the chromosomal region. Genes with CNVs in frequently altered chromosomal region were indicated.

**Fig. S6.** The R361H loss-of-function mutation in SMAD4 inactivates TGF-β/SMAD signaling in MBA-MD-468 cells. MBA-MD-468 cells were co-transfected with constructs encoding flag-tagged WT or mutant SMAD4, SBE4-Luc, and pRL-TK. After 24 h, cells were stimulated with or without 5 ng/ml TGF-β1 for 15 h. Cells were lysed and promoter activities were measured using a dual luciferase assay system. Firefly luciferase activities were normalized with respect to Renilla luciferase activities. Values on bar graphs were shown as mean ± SD of three independent experiments. Intergroup comparisons were conducted by using student’s t-test. (NS, not significant; ***, *p* < 0.001).
